# A Novel Hybrid Additive Manufacturing Process for Drug Delivery Systems with Locally Incorporated Drug Depots

**DOI:** 10.3390/pharmaceutics11120661

**Published:** 2019-12-07

**Authors:** Jan Konasch, Alexander Riess, Robert Mau, Michael Teske, Natalia Rekowska, Thomas Eickner, Niels Grabow, Hermann Seitz

**Affiliations:** 1 Microfluidics, Faculty of Mechanical Engineering and Marine Technology, University of Rostock, Justus-von-Liebig Weg 6, 18059 Rostock, Germany; jan.konasch@uni-rostock.de (J.K.); alexander.riess@uni-rostock.de (A.R.); robert.mau@uni-rostock.de (R.M.); 2 Institute for Biomedical Engineering, University Medical Center Rostock, Friedrich-Barnewitz-Straße 4, 18119 Rostock, Germany; michael.teske@uni-rostock.de (M.T.); natalia.rekowska@uni-rostock.de (N.R.); thomas.eickner@uni-rostock.de (T.E.); niels.grabow@uni-rostock.de (N.G.); 3 Department LL&M, Interdisciplinary Faculty, University of Rostock, Rostock, Albert-Einstein-Str. 25, 18059 Rostock, Germany

**Keywords:** drug delivery system, hybrid additive manufacturing process, stereolithography, inkjet printing, drug depot, time-controlled drug release, poly(ethylene glycol) diacrylate (PEGDA)

## Abstract

Here, we present a new hybrid additive manufacturing (AM) process to create drug delivery systems (DDSs) with selectively incorporated drug depots. The matrix of a DDS was generated by stereolithography (SLA), whereas the drug depots were loaded using inkjet printing. The novel AM process combining SLA with inkjet printing was successfully implemented in an existing SLA test setup. In the first studies, poly(ethylene glycol) diacrylate-based specimens with integrated depots were generated. As test liquids, blue and pink ink solutions were used. Furthermore, bovine serum albumin labeled with Coomassie blue dye as a model drug was successfully placed in a depot inside a DDS. The new hybrid AM process makes it possible to place several drugs independently of each other within the matrix. This allows adjustment of the release profiles of the drugs depending on the size as well as the position of the depots in the DDS.

## 1. Introduction

Personalized drug therapy offers great potential to optimize treatments for every patient [1]. However, conventional drug manufacturing techniques are limited in taking a patient’s individuality into account, which could result in inadequate treatment doses and/or adverse effects, which could be hazardous for pediatric and geriatric patient groups [2]. In recent years, additive manufacturing (AM), also referred to as 3D printing, has gained much attention for offering beneficial opportunities to overcome such limitations. In the medical field, AM techniques are powerful tools for custom-made solutions for surgical instruments, prosthetics, and implants that meet the individual anatomical needs of the patient [3,4,5,6,7]. Furthermore, the technology holds promising potential for tissue engineering and bioprinting as well as organ and disease modeling [8,9,10,11,12]. However, in the pharmaceutical field the technology is still at an early stage [1,5]. Thus, it took until the year 2015 for the first 3D-printed drug Spritam^®^ by Aprecia Pharmaceuticals (Blue Ash, OH, USA) to be approved by the United States Food and Drug Administration (FDA) [13]. Nevertheless, extensive research in recent years has shown immense opportunities in the creation of additive manufactured personalized medicines with respect to the patient’s individual characteristics such as age, weight, and pharmacogenetics [2]. In that context, multifunctional drug delivery systems (DDSs) with flexible drug formulations for increased bioavailability and accelerated release characteristics, especially for oral, oromucosal, and topical drug therapeutics, have been demonstrated [1,2,5].

AM is an umbrella term for a multitude of processes which differ in major parameters such as solidification process, materials, resolution, and printing speed. The main methods are powder solidification, liquid solidification, and extrusion [2,5]. The suitability of various AM processes for the creation of DDSs has been demonstrated, such as powder solidification via drop-on-powder-printing (DOP), also referred to as binder jetting (BJ), or selective laser sintering, and liquid solidification via stereolithography (SLA), as well as polyjet/multijet-printing and extrusion-based methods. The ZipDose^®^ method by Aprecia^®^ Pharmaceuticals that is used for Spritam^®^ is a prominent example of a DOP process in the pharmaceutical field. Nevertheless, it seems that extrusion-based methods such as fused deposition modelling (FDM) are being investigated the most, especially regarding multidrug DDSs [1,5]. The printing devices are compact, the equipment is relatively inexpensive, and the technique is suitable for multicomponent printing because of the relatively simple integration of multiple print heads on one device. However, extrusion methods generally need heating steps that are often not suitable for thermolabile active pharmaceutical ingredients (APIs). Moreover, compared to SLA the printing resolution of extrusion-based as well as powder-solidification-based printing methods is limited. In the SLA, process energy is transferred into a liquid photopolymerizable resin via light beam, e.g., a UV laser. Precise system optics allow high-resolution printing since the resolution is not limited by powder particle diameter or filament extrusion. For pharmaceutical applications it can be advantageous that localized heating is minimized during printing because of the low energy input [14]. This is beneficial especially for the processing of thermolabile APIs. In that context, the photopolymerization process should also not be critical in terms of thermal API degradation [1,15]. A key advantage of SLA is the capability of controlling the material properties via photopolymerization parameters, i.e., exposure parameters or crosslinking [16,17]. This also offers promising methods for API incorporation in the DDS to control release kinetics, especially for large-molecular-weight APIs such as proteins.

Usually, SLA devices operate with a single photopolymer. For the manufacturing of a DDS, a homogenous mixture of drug and photopolymer has to be prepared. For this reason, the targeted drug has to be soluble in the photopolymer. The SLA-printed DDS finally features a homogeneous API distribution in the polymer matrix which leads to a continuous release profile with an initial burst release of the drug. Due to those facts, SLA is limited with regard to multidrug as well as high drug-loading applications [1]. Basically, multi-material SLA is reported in literature as well, primarily based on techniques using multiple material vats [18,19,20,21]. However, frequent vat switching or high numbers of different materials are especially technically challenging for such systems [9].

Here we introduce a hybrid AM process that combines SLA with inkjet printing (IJP). IJP is a high-precision fluid dispensing technology for droplet sizes down to the picoliter scale. It is an easily scalable, highly automated technology with promising potentials for medicine formulation techniques [22] as well as medical and pharmaceutical printing applications [23,24]. The new hybrid AM method enables the generation of multidrug DDSs with precisely localized drug depots, which is very promising for time and direction-controlled drug release or somewhat delayed and long-term drug release. In detail, a DDS will be built layer-by-layer via a conventional SLA technique, while inkjet printheads are used to generate precise small fluid volumes of drug-solutions that will be incorporated or crosslinked with the polymer matrix of the DDS. This new method enables free positioning of local drug depots inside the DDS with defined size and diffusion paths to achieve time-controlled drug release.

## 2. Materials and Methods 

### 2.1. Hybrid Additive Manufacturing Process

#### 2.1.1. Technical Concept

The technical concept of the hybrid AM process consists of an SLA system that builds the base polymer matrix of the DDS and inkjet printheads which are used to incorporate drugs into depots in a polymer matrix. A preliminary approach as previously presented in [25] has to be further revised and a prototype system has to be realized. 

For the targeted hybrid AM system, an in-house developed SLA system [26] was used as a basis and was modified. Unlike laser-scanning SLA systems, this system uses a laser diode as an irradiation source and the focusing optics are moved by a pair of horizontal x–y axes. In order to realize the hybrid AM concept, a new optical unit consisting of a laser diode, a beam-forming unit, and a focusing unit had to be integrated in the existing SLA apparatus. The wavelength of the laser diode should correspond to the absorbance spectrum of the chosen photoinitiator lithium-phenyl-2,4,6-trimethylbenzoyl-phosphinate (LAP), which has an absorption wavelength below 410 nm. The waist diameter of the resulting beam was determined using the Edmund Optics Beam Profiler 4M (Edmund Optics GmbH, Mainz, Germany).

Furthermore, two single-nozzle inkjet printheads had to be selected and integrated into the SLA system in order to realize a DDS with different APIs. The printheads should be able to process a broad range of different solutions and eject droplets with a volume in the sub-nl range in order to realize drug depots in the sub-100 micrometer range. The technical implementation included the mechanical integration, electrical connection and the modification of the programming. The system itself and the necessary technical components were designed using the computer-aided design (CAD) system Solidworks (Dassault Systèmes, Vélizy-Villacoublay, France). The control interface was realized in MATLAB Appdesigner (The MathWorks Inc., Natick, MA, USA).

#### 2.1.2. Printing Strategy

The 3D model of the base structure of the DDS was sliced into several layers with a defined layer thickness. The cross-sections were saved into bitmap files. To generate 3D structures the SLA system irradiated the resin line-by-line in accordance with the pattern of the bitmap file of the corresponding layer [26]. White pixels in the bitmap file indicate where the laser diode was turned on. Black pixels indicate where the laser diode was turned off. After completing the layer, the platform was lowered by 2 mm to recoat the surface with resin. After lowering, the platform was then raised again to the original position minus the layer thickness of the new layer. These steps were repeated layer-by-layer until a build height was reached where a drug depot had to be created.

**Figure 1:**
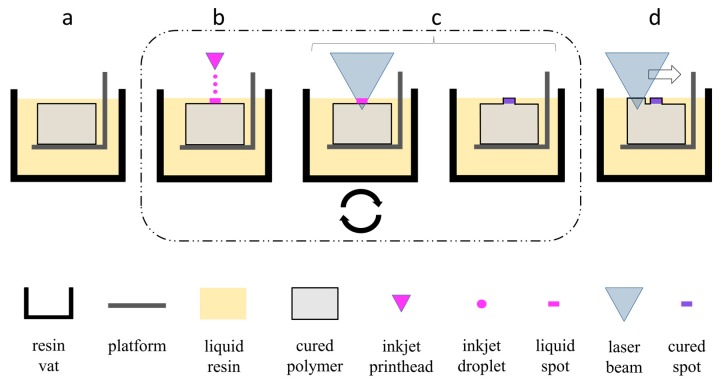
Schematic process flow for the incorporation of the drug spot (**a**) in the initial state, (**b**) spotting one or more drops of drug solution with an inkjet printhead on the desired location, (**c**) with curation and fixation of the spotted area, thereby creating a single drug spot, and (**d**) with line-by-line curing of base structure. The drug depot consisted of a stack of a defined number of single drug spots in consecutive layers (see Figure 2b).

The incorporation of drug depots using IJP during the SLA building process is depicted in Figure 1. After recoating, the inkjet printhead was positioned to the desired location of the drug depot. As soon as the position was reached, the inkjet printhead started dispensing a defined number of droplets of the drug solution. Right after dispensing the optic was placed above the spot and the laser diode was switched on for a defined period of time. The local irradiation led to curing of the spotted area and thus to a fixation of the applied material, thereby creating a single drug spot. This cycle was repeated for each depot in the corresponding layer. After all drug spots were placed and cured the SLA printing was continued by line-by-line curing as described above.

**Figure 2:**
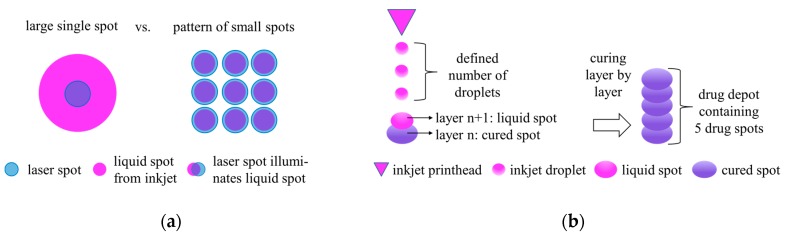
(**a**) Schematic illustration of the possibility of incorporating a high amount of active pharmaceutical ingredient (API) into the drug delivery system (DDS) by dividing one large singe spot into a spot pattern; (**b**) drug depot consisting of a stack of a defined number of single drug spots (or spot pattern) in consecutive layers.

When incorporating drug depots using IJP it is important that the printed liquid spot is stable on the liquid surface of the resin during the short period of time until the irradiation source is placed and cures the spot. Furthermore, the size of the liquid spot is limited by the laser spot that possibly has to fix the entire liquid spot. This limits the number of drops that can be placed in one spot and thus restricts the size of a single drug spot. To overcome this limitation, several independent spots could be placed next to each other (see Figure 2a). In case of creation of spot patterns, each spot of the pattern had to be irradiated immediately after inkjet printing before the next spot was created, which finally led to longer process times compared to the creation of single spots. An arrangement of several single drug spots or spot patterns over a defined number of layers finally led to a drug depot, as depicted in Figure 2b. A drug depot consisting of a stack of single spots resulted in a cylindrically-shaped drug depot, whereas in the case of a spot pattern, the shape of the drug depot depended on the arrangement of the spot pattern itself, leading e.g., to a cuboid-like depot. However, no sharp depot contours could actually be achieved due to the limited resolution of the printed spots and the initial diffusion of the spotted drug solution immediately after jetting.

In order to ensure the correct operation of the inkjet printheads a test routine had to be implemented. Before the loading of the drug depots starts, inkjet printheads were moved over a sheet of water-sensitive paper positioned next to the building platform to dispense up to five droplets each. This test was used to check if the printheads were reliably dispensing throughout the whole building process and to prevent drying and associated clogging of the fluid channel. 

### 2.2. Preliminary Experimental Study

#### 2.2.1. Photopolymer and Parameters for SLA Printing

In order to validate the technical concept, an initial experimental study had to be performed. The photopolymer poly(ethylene glycol) diacrylate (PEGDA, M_n_ = 700 g/mol, Sigma-Aldrich, Taufkirchen, Germany) was used, as it is considered to be biocompatible and is commonly used in a broad range of biomedical applications [15,27,28,29]. As a photoinitiator, lithium-phenyl-2,4,6-trimethylbenzoyl-phosphinate (LAP) with an absorption wavelength below 410 nm was used. For initial studies the chosen resins were pure PEGDA (M_n_ = 700 g/mol, Sigma-Aldrich), PEGDA mixed with ultrapure water (4:1), and PEGDA mixed with ultrapure water (1:1). Based on the mass fraction of the polymer 0.07% (*w*/*w*) of LAP was added to every PEGDA resin. The PEGDA resins as well as the local drug depots were irradiated by a laser diode with a peak wavelength of λ = 405 nm and a nominal power of P = 20 mW and a focus diameter of d = 90 µm.

#### 2.2.2. Test Liquids for IJP

For initial tests a blue ink (4001 TP/6 Königsblau) and a pink ink (4001 TP/6 Pink), both from Pelikan, Feusisberg, Switzerland, were used to visualize the resulting depots. The content of 0.8 mL of each ink cartridge was filtered with a syringe filter (ACRODISC PSF Versapor 0.8 µm, PALL, Port Washington, NY, USA) and diluted in 16 mL of ultrapure water. The resulting ink solutions for the blue and pink ink were applied for different printing tests. Additionally, bovine serum albumin (BSA) (Sigma-Aldrich) labeled with Coomassie blue (Applichem GmbH, Darmstadt, Germany) was used to generate first depots with a model drug. A BSA solution of glycerol and formic acid (1:1) containing 5 mg/mL BSA labeled with Coomassie blue (0.3 mg/mL) was prepared.

The actual volume of an ejected droplet was determined optically using the stroboscope camera of the micro-dispensing device Nanoplotter 2.1 (GeSiM, Gesellschaft für Silizium-Mikrosysteme mbH, Radeberg, Germany) for each test liquid. Each measurement was repeated three times.

#### 2.2.3. Single and Multiple Depots

To demonstrate and evaluate the possibilities of the process, first, samples were generated. As the geometry for the specimen, a cuboid with an edge length in x–y direction of s_xy_ = 4 mm and a height of s_z_ = 5.5 mm was chosen. The layer thickness was z = 0.1 mm and the linefeed was 50 µm. The travel speed of the laser in x-direction was v = 30 mm/s. Irradiation time for inkjet-spots after placement was t_irr_ = 0.5 s. As a resin, PEGDA mixed with ultrapure water (1:1) was used.

**Figure 3:**
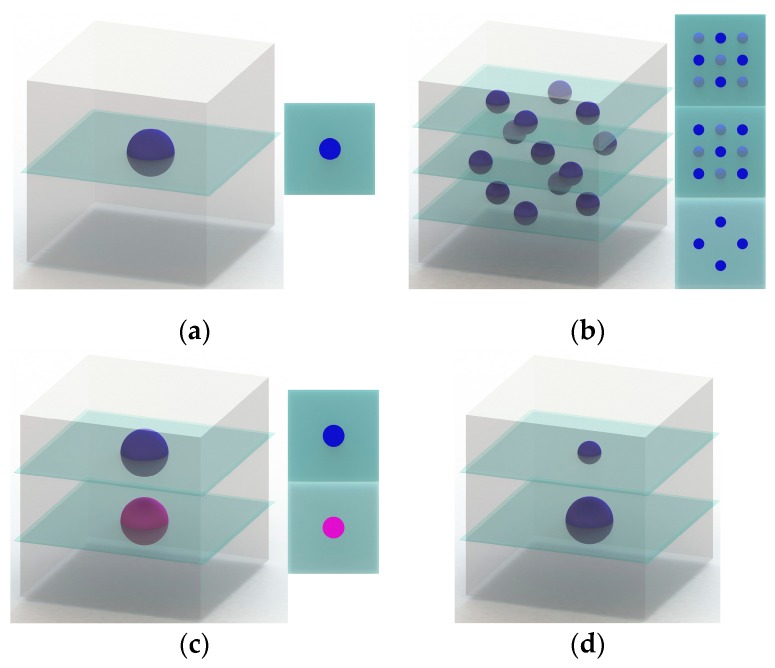
Schematic illustration of the different test specimens: (**a**) specimen with one single depot (blue ink); (**b**) specimen with 13 individual depots over three planes (blue ink), sectional views of corresponding planes; (**c**) specimen with blue and pink ink depots placed with two printheads in two separate planes; (**d**) specimen for the long-term diffusion study with two differently sized ink depots (blue ink).

The first specimen contains one single blue depot (see Figure 3a). The depot consisted of five spots built over five consecutive layers. In each spot five drops of the blue ink solution were dispensed.

The second specimen contained 13 individual blue depots over three planes (number of depots in order from bottom to top: 4, 5, 4; see Figure 3b). Again, each depot consisted of five spots built over five consecutive layers. In each spot five drops of the blue ink solution were dispensed. The distance between spotting planes was approximately 1 mm.

The third specimen featured two single depots loaded with two different inks (blue and pink) using both inkjet printheads. Each depot was generated in the same manner as the single depot described above. Five spots were built over five consecutive layers. Five drops of the respective ink solution are dispensed in each spot.

#### 2.2.4. Long-Term Diffusion Study

To qualitatively investigate the diffusion of the ink in the PEGDA base structure over time, a specimen with two depots (one over five layers, the second over 10 layers, one spot per layer, five drops of the blue ink solution per spot) was generated. As the geometry for the specimen, a cube with an edge length of s_xyz_ = 4 mm was chosen. The layer was z = 0.1 mm and the linefeed was 50 µm. The travel speed in the x-direction was v = 40 mm/s. Irradiation time for inkjet spots after placement was t_irr_ = 0.5 s.

As a resin, PEGDA mixed with ultrapure water (4:1) was used. The depots were made from blue ink. The schematic illustration of the specimen is shown in Figure 3d. For comparison two specimens were built. One specimen was stored in ultrapure water for 14 days, while another one was stored in a sample bag at room temperature.

The length in x–y direction and the height of both specimens was measured three times after 14 days of storage in water and the sample bag, respectively, using a digital caliper (resolution 0.01 mm, accuracy ± 0.02 mm) in order to determine the dimensional changes due to the swelling of the hydrogel base structure. The dimensions were determined by the averaging of three measured values.

#### 2.2.5. DDS with Incorporated Model Drug Depot

To generate a simple DDS with a central model drug depot, BSA labelled with Coomassie blue was used. As the geometry for the specimen, a cube with an edge length of s_xyz_ = 4 mm was chosen. The layer thickness was z = 0.1 mm and the linefeed was 50 µm. The travel speed in the x-direction was v = 40 mm/s. The irradiation time for inkjet-spots after placement was t_irr_ = 0.5 s. As a resin, pure PEGDA was used. The depot was built over five layers containing one spot per layer at which each spot consisted of five drops of the BSA solution. 

## 3. Results

### 3.1. Hybrid Additive Manufacturing System

**Figure 4:**
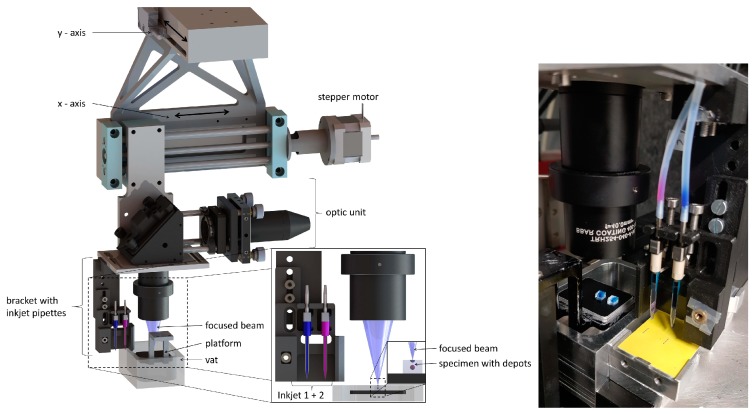
Left: Computer-aided design (CAD) scheme of the hybrid additive manufacturing system; Right: prototype of hybrid additive manufacturing (AM) system based on stereolithography (SLA) combined with inkjet printing (IJP); the yellow water-sensitive paper was used to check the correct operation of the IJPs.

A CAD model of the hybrid AM system and the assembled experimental setup are depicted in Figure 4. The optical unit was mounted on horizontal x–y axes driven by stepper motors. While the x-axis (high helix lead screw module, igus GmbH, Cologne, Germany) was used as a scanning axis and therefore needed to be moved quickly, the linefeed was done by a slowly moving y-axis (PLS85 4”, PI miCos GmbH, Eschbach, Germany). As inkjet printheads, two single-nozzle Nano-Tip J piezoelectric pipetting tips from GeSiM (Gesellschaft für Silizium-Mikrosysteme mbH, Radeberg, Germany) were chosen for integration into the SLA system. The printheads were driven at a voltage of U = 100 V, a pulse width of t_p_ = 50 µs and a frequency of f = 100 Hz. The printheads were mechanically connected to the carrier of the optic unit using a bracket. The bracket also served as mechanical protection. The distance of the printhead tips to the upper surface of the vat was adjusted to 1.5 mm. The printheads were filled with the drug or ink solutions by a syringe via a flexible tube. Water-sensitive paper could be positioned on a platform next to the vat (yellow paper in Figure 4 right). The inkjet printheads were moved over the sheet and printed up to five droplets on the paper prior to every depot loading step for every layer. 

**Figure 5:**
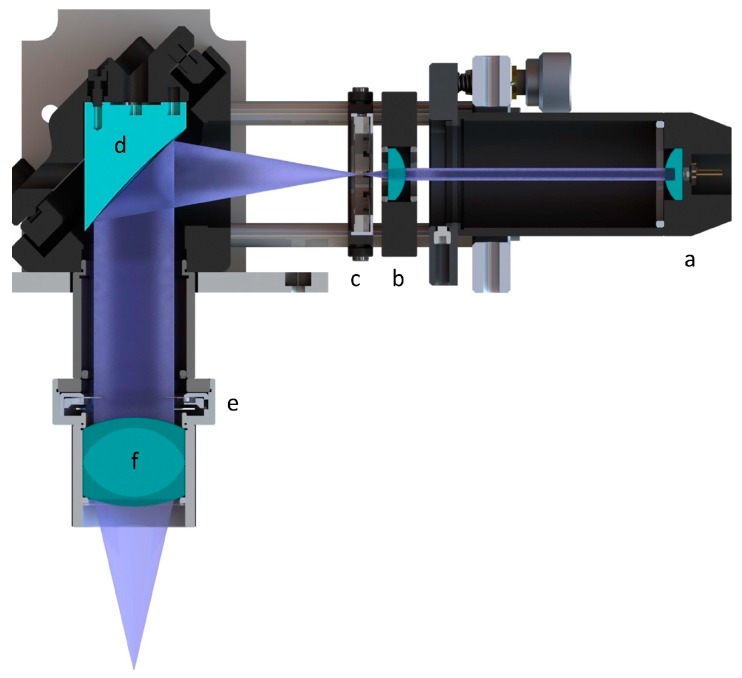
Cross-section of the new optic unit; (**a**) laser diode (with aspheric lens); (**b**) plan-convex lens; (**c**) iris; (**d**) off-axis parabolic mirror; (**e**) iris; and (**f**) Hastings achromatic triplet.

A new optical unit was developed and integrated in the existing SLA setup. As an irradiation source, a laser diode (L405P20, Thorlabs, Newton, NJ, USA) with a wavelength of λ = 405 nm and power of P = 20 mW was chosen. The laser diode beam was collimated by an aspheric lens (C671TME-405, Thorlabs) as shown in Figure 5a. A positive meniscus lens (Figure 5b, LE5838, Thorlabs) in combination with an off-axis parabolic mirror (Figure 5d, MPD129-F01, Thorlabs) enlarged and redirected the collimated beam towards the vat. The focusing of the laser beam was achieved by using a Hastings triplet (Figure 5f, TRH254-040-A-ML, Thorlabs). After adjusting the optical elements, the resulting focal diameter was determined to be about d = 90 µm.

The optical unit and the inkjet pipetting tips were successfully installed in the SL-apparatus. The control electronics were created using a C2000 microcontroller (Texas Instruments, Sherman, TX, USA). The process flow was programmed in Code Composer Studio (Texas Instruments). The printing strategy of the hybrid manufacturing process was successfully implemented in the control interface. 

### 3.2. Experimental Studies

All PEDGA specimens were built successfully. All parts were removed from the platform and placed on a paper towel for drying. No further thermal or UV post-curing treatment was carried out. The actual dimensions of the cuboids closely correspond to the nominal values of s_xy_ = 4 mm and a height of s_z_ = 4 mm and s_z_ = 5.5 mm, respectively. The top surfaces of the specimens feature a slight ripple structure caused by the line-wise hardening process whereas the bottom surface show minor irregularities due to the mechanical removal of the parts from the building platform. 

The correct operation of the inkjet printheads for all tests could be verified by the test routine where droplets of the solution are printed on water-sensitive paper before loading the depots at every single layer. The measured drop volume was 786 pl ± 5 pl for the blue ink solution and 821 pl ± 22 pl for the pink ink solution. 

**Figure 6:**
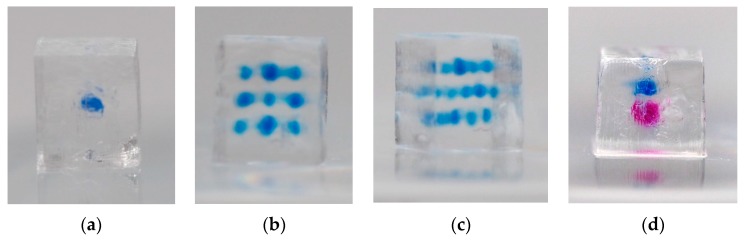
Poly(ethylene glycol) diacrylate (PEGDA)-based test specimens with different depots: (**a**) side view of the specimen with single drug depot containing 19.7 nl of blue ink solution; (**b**) top view and (**c**) angular view of the specimen with 13 individual depots containing a total of 256.1 nl blue ink solution; (**d**) side view of the specimen with two different ink depots containing 19.7 nl of blue and 20.5 nl of pink ink solution. The depots were created using two inkjet printheads.

The specimen with one single depot featured a clearly visible single blue ink depot (see Figure 6a). No major blurring could be observed. This test basically demonstrates the creation of a single depot with relatively sharp contours containing a total of 19.7 nl of blue ink solution within a PEGDA base structure. The specimen with multiple ink depots was built successfully (Figure 6b, top view, and 6c, angular view). All depots were clearly visible. Blurring could be observed. The blurring effect was increased optically due to the overlay of depots of the different planes. Four depots in the top view of Figure 6b appear larger than the others due to the direct overlay of depots in first and the third plane (compare with Figure 3b). Every single depot contained 19.7 nl of blue ink solution, leading to a total ink solution volume of 256.1 nl for all depots. The third test demonstrates successfully (see Figure 6d) that two different ink depots can be implemented in one single PEGDA base structure. Both ink depots can be clearly distinguished. The depots contained 19.7 nl of blue and 20.5 nl of pink ink solution, respectively. A higher blurring effect could be observed for the pink ink depot, leading to the impression of a larger depot volume.

**Figure 7:**
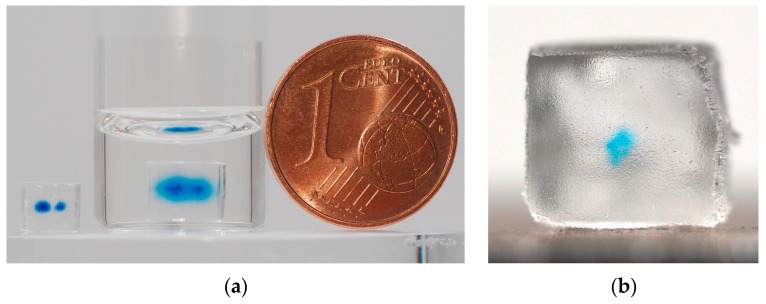
(**a**) Side view of the Poly(ethylene glycol) diacrylate (PEGDA)-based specimens with two differently sized depots containing 19.7 nl and 39.4 nl of blue ink solution after 14 days of storage (left specimen: dry storage in sample bag at room temperature, right specimen: storage in ultrapure water at room temperature); (**b**) side view of the PEGDA-based drug delivery system (DDS) with a central depot containing 55.65 ng of Coomassie blue-labeled bovine serum albumin (BSA).

The specimens of the long-term diffusion study are depicted in Figure 7a. Both specimens are pictured after 14 days of storage at room temperature, one in pure water and one in a bag with air. The two depots are clearly visible and it is noticeable that one depot is larger than the other one. The smaller depot contained 19.7 nl of blue ink solution, whereas the larger one contained 39.4 nl. The blurring of the small depot was comparable to blurring of the single depot in Figure 6a which contained the same volume of ink. This demonstrates that there was no major change of the depots during the time in which the samples were stored under dry conditions in the sample bag at room temperature. Conversely, the diffusion of the blue ink solution can be clearly noticed in the water-stored sample. The blue ink has nearly reached the surface area of the structure after 14 days. The depots are still centrally located in the base structure.

The long-term storage in water caused a swelling of the PEGDA base structure which is typical for hydrogel materials. The geometrical properties, volume, and mass of the specimen with multiple ink depots are shown in Table 1. A minor deviation of the measured dimensions of the sample stored in the sample bag from the nominal dimensions can be observed. The dimensional deviations are mainly caused by the limited accuracy SLA process. The results also show that the swelling after storage in water was nearly isotropic with linear expansion of about 18% in all directions leading to a volume increase of 66.5%.

A first PEGDA-based DDS with a depot of Coomassie blue-labeled BSA was successfully built. The specimen is depicted in Figure 7b. The depot is clearly visible. The measured drop volume for the BSA solution was 450 pl ± 37 pl. The depot contained 11.3 nl of BSA solution which corresponds to a loading of the depot with 55.65 ng of pure BSA. The saturation of the color is not as high as those of the depots containing ink solutions. No major blurring can be observed.

## 4. Discussion

First of all, the results show that the assembly and operation of the novel hybrid AM system based on an existing SLA setup [26] was successful. The system was running steadily during the experimental studies. The control software was able to run a stable automated SLA process. However, the locations for the depots must currently be programmed directly in the control software because the drug depot cannot be included in the STL data file of the DDS due to limitations of the STL file format. For the future it is planned to model the whole DDS with depots and export them using the Additive Manufacturing File (AMF) format. The AMF file format overcomes the limitations of the STL file format regarding the definition of multiple materials in one model and finally allows modelling DDSs with incorporated drug depots in one single file [30].

The process flow was stable and the experimental study delivered repeatable results regarding the generation of the base structure using the laser system and the generation of depots using the inkjet printheads. PEGDA is suitable as a base material for the hybrid AM process. It is possible to process PEGDA solutions with different water contents in an SLA process as it has already been demonstrated using other polymerization techniques [31,32]. The contour sharpness of the samples is good, especially for the resins with lower viscosities (PEGDA mixed with ultrapure water). Minor process-specific surface structures and irregularities can be detected. 

LAP has proven to be an appropriate photoinitiator for the PEGDA monomers that are processed in the hybrid AM process. Firstly, because LAP is one of the most successfully used photoinitiators for biocompatible stereolithography [9,29]. Secondly, LAP has a relatively high water solubility and allows for polymerization with visible light (near-UV blue light) which finally leads to a reduced impact on the API compared to photoiniators such as Irgacure^®^ 2959 (BASF SE, Ludwigshafen, Germany) which need UV light for polymerization [33,34]. Even if previous studies showed that UV photopolymerization in an SLA process did not lead to significant drug degradation [1], damage of specific APIs by UV light is still very likely. 

The chosen concentration of the photoinitiator LAP of 0.07% (*w*/*w*) was relatively low compared to concentrations described in other studies [35,36,37]. Further adjustment of the concentration could be made with regard to further improvement of biocompatibility or adjustment of crosslinking of the PEGDA base structure for defined diffusion or swelling behavior as well as reduced initial blurring of the depots. In this case, the corresponding SLA process parameters such as the travel speed of the laser spot or the light intensity must be adjusted. 

A typical swelling behavior of the PEGDA-based hydrogel structure could be observed for the specimen stored in water. Hydrogels are a three-dimensional polymer network which have the ability to absorb and retain water which finally causes swelling. The swelling has to be kept in mind when designing a DDS for a specific application. The swelling behavior, but also the mechanical properties as well as diffusive properties of hydrogels, can be tuned by varying the crosslink density making themselves optimal tools for various kinds of DDSs [38].

The experimental results show that it is possible to selectively incorporate fluids into depots inside a polymeric 3D structure generated by SLA. It could be demonstrated that in a sample several different depots, placed by different inkjet printheads, can be incorporated. A minor blurring of the depots occurred, which is likely to have been caused by the diffusion of the ink during the time gap between printing of the drug depot and the curing via laser light, which is currently in the range of some seconds. The time gap and consequently the blurring effect can be further reduced by optimized process strategies or suitable combinations of base polymer and API solution.

The long-term diffusion study demonstrated that an initial burst release of the ink stored in the depot, which is typical for SLA printed DDS [14,15], can be avoided. A delayed drug release can be achieved depending on the distance of the depot to the surface of the DDS. The ink has not reached the surface of the base structure after 14 days of storage in water. The diffusion of the ink was relatively slow. Since this experiment was performed with ink in order to obtain an optical impression of the diffusion behavior, no further analysis has been performed. The successful printing of the DDS with an incorporated BSA depot finally demonstrated that the technique can process solutions with APIs. The relatively low blurring of the BSA depot compared to the ink depots is plausible since the ink depots were loaded with a higher amount of solution and since the concentration of dye was much higher in the ink depots. Furthermore, pure PEGDA was chosen as the base material for the DDS, which probably leads to a reduced diffusion compared to PEGDA mixed with ultrapure water. Additionally, the relatively low blurring could also be caused by crosslinking of BSA with PEGDA by side thiol groups.

Future investigations will focus on a deeper understanding of the diffusion of concrete APIs which is basically determined by the kind of API, its position in the DDS, the base material, and crosslinking parameters. With this knowledge, it will then be possible to design a DDS with controlled release behavior for specific APIs. Furthermore, an increased dimensional accuracy can be achieved by further optimization of the SLA process parameters. 

The novel hybrid AM system enables the possibility of producing DDSs with differently incorporated APIs for multiple drug release without the need of changing the vat of the stereolithography system as it has been described previously [18,19,20,21]. Moreover, inkjet printing is an appropriate technology to process kinds of APIs, offering precise control in terms of drug formulation and delivering quantity [22,23,39]. This finally opens a broad field of application for the new hybrid AM process regarding the generation of DDSs. A preferred field of application is that of implantable biodegradable DDSs for multi-phase drug release for therapeutic or regenerative use. For example, the novel hybrid AM system allows the manufacturing of DDSs containing a first antiseptic drug that needs an initial rapid release, a second anti-inflammatory drug with medium-term release kinetics, and finally a third therapeutic or regenerative drug for long-term release. By localizing the drug depots in the implant as well as specific crosslinking of APIs with a PEGDA base structure, different release periods over a broad range in one single DDS can be achieved. 

## 5. Conclusions

The present study demonstrates the implementation of a new technical approach for a hybrid AM process for producing DDSs as well as a preliminary experimental result demonstrating the proof-of-concept. This hybrid AM process was able to create DDSs with drug depots of different diffusion distances inside the structure to achieve specific drug release kinetics. This new approach can overcome the limitations of classic SLA processes where DDSs have a homogeneous distribution of the API inside the polymer matrix.

Even if the proof-of-concept was successful, there is still a long way to go towards concrete DDSs. Further investigations will focus on the adjustment of the material parameters to be able to control the crosslinking in order to adjust the permeation, the swelling, and the technical parameters of the PEGDA base structure. It is necessary to investigate how the repeated exposure with light affects the effectiveness of the incorporated APIs. Drug release studies are necessary to investigate the time-dependent release of drugs from the DDS reliant on the chosen drug solution, base material, and processing parameters. A deeper understanding of the process can finally lead to the development of DDSs with time-controlled drug release, combined drug products with multiple APIs, or specific medicinal products tailored to an individual patient.

## 6. Patents

The device and the method described in this article are protected by a German patent DE102018107585B3 „Vorrichtung zur Herstellung von 3D-gedruckten Wirkstofffreisetzungssystemen mit Wirkstoffdepots, sowie Verfahren zur Herstellung von 3D-gedruckten Wirkstofffreisetzungssystemen“ (apparatus for the production of 3D printed drug delivery devices with drug depots as well as the method for the production of 3D printed drug delivery devices) and, in addition, an international PCT patent application on this topic has been filed recently.

## Figures and Tables

**Table 1:** Geometrical properties and volume of the specimens with multiple ink depots.

Parameter	Nominal Value	Dry Storage	Storage in Water
s_x_ (mm)	4	4.1 ± 0.14	4.85 ± 0.16
s_y_ (mm)	4	3.93 ± 0.036	4.66 ± 0.027
s_z_ (mm)	4	4.33 ± 0.038	5.14 ± 0.03
Volume (mm^3^)	64	69.77	116.17
